# Laparoscopic extraperitoneal techniques in ventral hernia repair: a retrospective comparative study of TAPP and TEP

**DOI:** 10.3389/jaws.2026.15676

**Published:** 2026-05-08

**Authors:** R. Croceri, A. Tek, L. Aragone, J. P. Medina, D. E. Pirchi

**Affiliations:** Abdominal Wall Unit, General Surgery Department, British Hospital of Buenos Aires, Buenos Aires, Argentina

**Keywords:** extraperitoneal mesh placement, laparoscopic, TAPP, TEP, ventral hernia repair

## Abstract

**Background:**

Ventral hernia repair has traditionally been performed laparoscopically using the intraperitoneal onlay mesh (IPOM) technique, which is simple and widely available but carries the drawback of mesh–viscera contact. Extraperitoneal approaches such as transabdominal preperitoneal (TAPP) and totally extraperitoneal (TEP) repair have emerged as alternatives that avoid this limitation, allowing the use of standard meshes without fixation. However, evidence comparing these techniques remains scarce.

**Methods:**

We conducted a retrospective comparative analysis of a prospectively collected database at a single centre between January 2023 and December 2024. Patients undergoing laparoscopic repair of primary ventral or W1 incisional hernias smaller than 4 cm, with or without rectus diastasis, were included. Demographic data, operative details, and postoperative outcomes were recorded. Pain was assessed using the visual analogue scale (VAS). Complications were graded according to Clavien–Dindo. Follow-up was at least 12 (twelve) months for all patients.

**Results:**

Thirty-three patients were analysed, 18 in the TEP group and 15 in the TAPP group. Baseline demographic and comorbidity characteristics were comparable. Operative time was significantly longer for TAPP (96.1 ± 14.8 vs. 84.7 ± 13.1 min, p=0.029). Mesh area was larger in TEP (242.9 ± 110.3 vs. 166.9 ± 49.9 cm^2^, p=0.015). Rectus diastasis was present in a similar proportion of patients (55.6% vs. 53.3%), but correction was achieved only in TEP (11 of 18 cases, 61.1%). There was one conversion to IPOM in TAPP. Postoperative pain scores, hospital stay (18.2 vs. 15.3 h, p=0.119), and 30-day complication rates (13.3% vs. 22.2%, p = 0.665) were comparable, consisting mainly of minor seromas or haematomas. No recurrences were observed at 1 year.

**Conclusion:**

Both TAPP and TEP are safe and effective minimally invasive approaches for small ventral and W1 incisional hernia repair. TAPP is associated with longer operative times due to peritoneal flap creation, while TEP enables broader mesh placement and ergonomic correction of rectus diastasis. Despite the small sample size and retrospective design, our findings add to the growing evidence supporting extraperitoneal approaches as valuable alternatives to IPOM in abdominal wall surgery.

## Introduction

Ventral hernias are among the most common abdominal wall pathologies, and their repair represents a relevant clinical and healthcare challenge. Surgical treatment has evolved with the aim of reducing recurrence, postoperative complications, and associated costs, driving the development of various minimally invasive techniques to optimize clinical and functional outcomes [1].

In this context, extraperitoneal approaches such as transabdominal preperitoneal (TAPP) and totally extraperitoneal variants—including the endoscopic totally extraperitoneal approach (TEA) [2], the subxiphoid top-down endoscopic totally preperitoneal approach (eTPA) [3], and the enhanced-view totally extraperitoneal technique (eTEP) [4]—have been developed. These allow the use of standard meshes without fixation and avoid contact between the prosthesis and the abdominal cavity, showing encouraging results in terms of safety, costs, and postoperative comfort [2–5]. Nevertheless, scientific evidence supporting these extraperitoneal approaches remains limited and largely comes from early series and observational studies [2–5].

This study aims to analyze and compare the outcomes of laparoscopic ventral hernia repair using the transabdominal preperitoneal (TAPP) technique versus a ventral totally extraperitoneal approach performed using a subxiphoid top-down access (eTPA). The purpose is to provide updated evidence to assess the advantages and limitations of each strategy and contribute to the optimization of minimally invasive ventral hernia repair.

## Materials and methods

A retrospective comparative study based on a prospective database was carried out in a private community hospital between January 2023 and December 2024.

### Inclusion and exclusion criteria

All patients undergoing laparoscopic repair of primary or incisional ventral hernia with defects smaller than 4 cm (classified as small and medium in primary hernias and W1 of the EHS classification in incisional hernias), with or without rectus diastasis, and with a body mass index (BMI) greater than 25 kg/m^2^ were included. Patients with defects greater than 4 cm, previous mesh repairs, urgent or strangulated cases, loss of follow-up, and patients with rectus diastasis greater than 4 cm (treated with a different technique, eTEP Rives-Stoppa) were excluded.

### Variables

Demographic variables included age, sex, BMI, previous abdominal surgery, comorbidities, and physical status according to the ASA classification.

Intraoperative variables included: type of hernia (primary or incisional), defect size (EHS classification), defect location, operative time in minutes, presence of rectus diastasis and whether it was treated by plication, prosthesis size expressed in cm^2^, and the need for conversion.

Postoperative variables included: pain assessed with the visual analogue scale (VAS) immediately, on day 7, and on day 30; complications within the first 30 days, classified according to Clavien–Dindo; length of hospital stay in hours; and clinical recurrence at 1 year of follow-up. Minimum follow-up was 12 months, completed by 100% of patients.

### Patient allocation and surgical approach selection

Patient allocation to TAPP or TEP was not randomized and reflected the evolution of our surgical practice over time. During the initial period of the study, the TAPP approach was preferentially used. With increasing experience and the adoption of extraperitoneal techniques, patients with the same clinical indications were progressively treated using a ventral TEP approach. This transition was driven by favourable early results regarding operative time, mesh coverage, and the possibility of midline reconstruction, while maintaining consistent inclusion criteria throughout the study period.

### Statistical analysis

Quantitative variables were expressed as mean and standard deviation (SD) or median and interquartile range (IQR), according to distribution. Categorical variables were presented as absolute frequencies and percentages. For group comparisons, Student’s t-test or Mann–Whitney U test were used for continuous variables, and chi-square or Fisher’s exact test for categorical variables. A p-value <0.05 was considered significant. Statistical analysis was performed using SPSS 13.0 (SPSS Inc., Chicago, IL, USA).

### Ethical approval

This study was reviewed and approved by the Institutional Review Board (IRB), and written informed consent was waived by the IRB owing to the study’s retrospective nature.

### Surgical techniques

#### Ventral TAPP

**FIGURE 1:**
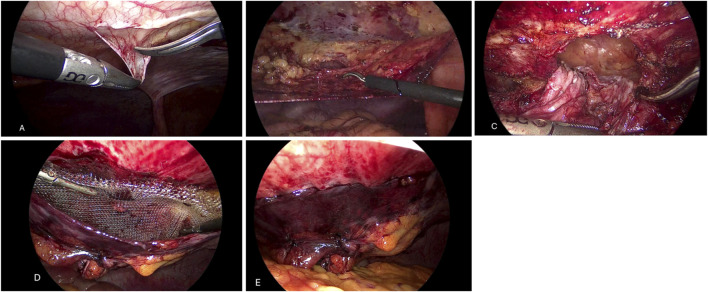
TAPP approach. **(A)** Peritoneal incision. **(B)** Peritoneal flap. **(C)** Reduction of hernia sac. **(D)** Mesh placement. **(E)** Peritoneal closure.

This approach begins with the creation of pneumoperitoneum using a Veress needle placed at Palmer’s point. Subsequently, access under direct vision is made through the left flank, with two additional 5-mm trocars placed. After completing the initial exploration, a parietal peritoneum incision is performed with hook or scissors from the left hypochondrium to the iliac fossa, about 6 cm lateral to the midline. A peritoneal flap is then created to expose the defect. The hernia sac is carefully reduced, and the defect is closed with a 0 absorbable barbed suture. An appropriately sized mesh is placed in the preperitoneal space without additional fixation. Finally, the peritoneal flap is closed with a 3-0 absorbable barbed suture, ensuring complete coverage of the prosthesis and avoiding contact with the viscera (Figure 1).

#### Ventral TEP

Totally extraperitoneal ventral hernia repair includes several technical variants based on the creation of a preperitoneal working space without entering the abdominal cavity. These comprise the endoscopic totally extraperitoneal approach (TEA) [2], the subxiphoid top-down endoscopic totally preperitoneal approach (eTPA) [3], and PeTEP (preperitoneal extended-view totally extraperitoneal approach) [5]. Although these techniques differ mainly in the access site and direction of dissection, they share the same extraperitoneal anatomical principles.

In this series, all ventral TEP procedures were performed using a subxiphoid top-down endoscopic totally preperitoneal approach (eTPA). The surgeon was positioned at the head of the patient, with the vision tower placed directly in front of the operator.

After initial access, a small incision and digital dissection of the preperitoneal space were performed, followed by trocar placement under direct vision. Dissection was carried out between the peritoneum and the posterior rectus sheath. The hernia sac and its contents were reduced, and the defect was closed using a continuous absorbable barbed suture. When rectus diastasis was present, it was incorporated into the midline closure, allowing simultaneous correction.

**FIGURE 2:**
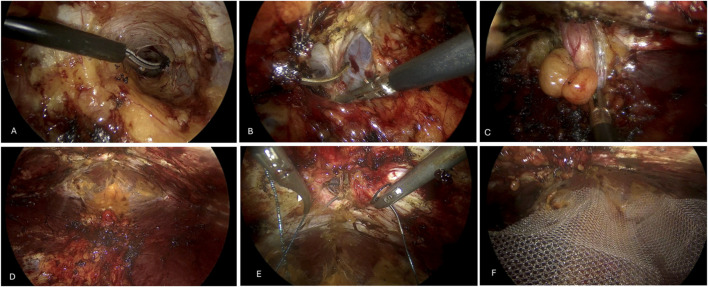
TEP approach. **(A)** preperitoneal space. **(B)** Dissection of the transversalis fascia and entry into the right pretransversalis space. **(C)** Reduction of the umbilical sac. **(D)** Completed dissection. **(E)** Defect closure. **(F)** Mesh placement.

A standard macroporous polypropylene mesh was then inserted and adapted to the preperitoneal space with at least a 5-cm overlap. No mesh fixation was used, as mesh stability was ensured by pneumopreperitoneum pressure and closure of the preperitoneal space (Figure 2).

## Results

Thirty-three patients were analysed, 18 in the TEP group and 15 in the TAPP group. Demographic and comorbidity characteristics are shown in Table 1. Both groups were comparable regarding age (50.3 ± 13.0 vs. 53.1 ± 12.3 years; p=0.540), sex (61.1% vs. 60% male; p=0.940), and BMI (30.3 ± 3.4 vs. 28.7 ± 2.7 kg/m^2^; p=0.148). Distribution of comorbidities was similar, with no significant differences in hypertension, diabetes, dyslipidaemia, smoking, or ischaemic heart disease.

**TABLE 1:** Demographic characteristics and comorbidities according to surgical technique.

Variable	TEP (n=18)	TAPP (n=15)	*p*
Age, years (mean ± SD)	50.3 ± 13.0	53.1 ± 12.3	0.54
Male sex, n (%)	11 (61.1%)	9 (60.0%)	0.94
BMI, kg/m^2^ (mean ± SD)	30.3 ± 3.4	28.7 ± 2.7	0.148
ASA II, n (%)	17 (94.4%)	13 (86.7%)	0.86
ASA III, n (%)	1 (5.6%)	2 (13.3%)	0.86
Hypertension, n (%)	4 (22.2%)	1 (6.7%)	0.346
Type 2 diabetes, n (%)	2 (11.1%)	2 (13.3%)	1
Dyslipidemia, n (%)	4 (22.2%)	3 (20.0%)	1
Ischemic heart disease, n (%)	4 (22.2%)	1 (6.7%)	0.346
Smoking, n (%)	4 (22.2%)	3 (20.0%)	1

BMI: body mass index, ASA: american society of anesthesiologists score.

Regarding operative variables (Table 2), the proportion of primary and incisional hernias was equivalent. In primary hernias, small defects W1 (0–2 cm) predominated, while all incisional hernias corresponded to W1 (0–4 cm) according to EHS. Operative time was longer in the TAPP group (96.1 ± 14.8 min; 95% CI: 87.8–104.3) compared to TEP (84.7 ± 13.1 min; 95% CI: 78.2–91.3; p=0.029). Mesh area was larger in TEP (242.9 ± 110.3 cm^2^; 95% CI: 188.1–297.8) than in TAPP (166.9 ± 49.9 cm^2^; 95% CI: 139.2–194.5; p=0.015).

**TABLE 2:** Operative characteristics according to surgical technique.

Variable	TEP (n=18)	TAPP (n=15)	*p*
Hernia type	​	​	1
– Primary, n (%)	11 (61.1%)	9 (60.0%)	​
– Incisional, n (%)	7 (38.9%)	6 (40.0%)	​
Defect size – Primary	​	​	1
W1 (0–2 cm), n (%)	8 (72.7%)	6 (66.7%)	​
W2 (2–4 cm), n (%)	3 (27.3%)	3 (33.3%)	​
Defect size – Incisional W1 (EHS Clasification)	7 (100%)	6 (100%)	—
Operative time, min (mean ± SD)	84.7 ± 13.1	96.1 ± 14.8	0.029
Mesh area, cm^2^ (mean ± SD)	242.9 ± 110.3	166.9 ± 49.9	0.015
Patients with diastasis	10 (55.6%)	8 (53.3%)	1
Diastasis treatment, n (%)	10 (55.6%)	0 (0%)	<0.001
Conversions, n (%)	0 (0%)	1 (5.6%)	1

EHS: european hernia society.

Rectus diastasis occurred with similar frequency (55.6% in TEP vs. 53.3% in TAPP). However, only patients in the TEP group underwent diastasis plication, which was performed in 11 of the 18 cases (61.1%) (p < 0.001). No plications were performed in TAPP. There was one conversion to IPOM in the TAPP group due to thin peritoneum, with no conversions in TEP.

Postoperative results are detailed in Table 3. No significant differences were observed in postoperative pain, either immediate, at day 7, or at 30 days. Length of stay was slightly longer in TAPP (18.2 ± 5.7 vs. 15.3 ± 4.1 h; p=0.119), without statistical significance. The overall complication rate was low and similar (22.2% in TEP vs. 13.3% in TAPP; p=0.665), mainly mild seromas and haematomas (Clavien–Dindo I). No recurrences were observed at 1-year follow-up in either group.

**TABLE 3:** Postoperative outcomes according to surgical technique.

Variable	TEP (n=18)	TAPP (n=15)	*p*
Immediate VAS pain (mean ± SD)	3.9 ± 1.5	4.0 ± 0.9	0.898
Day-7 VAS pain (mean ± SD)	1.9 ± 0.6	2.2 ± 0.8	0.211
Day-30 VAS pain (mean ± SD)	1.3 ± 0.5	1.5 ± 0.6	0.6
Length of hospital stay, hours (mean ± SD)	15.3 ± 4.1	18.2 ± 5.7	0.119
30-day complications, n (%)	4 (22.2%)	2 (13.3%)	0.665
– Seroma	3 (16.7%)	1 (6.7%)	—
– Hematoma	1 (5.6%)	1 (6.7%)	—
1-year recurrence, n (%)	0 (0%)	0 (0%)	—

## Discussion

This study compared two laparoscopic extraperitoneal approaches for ventral hernia repair: transabdominal preperitoneal (TAPP) and totally extraperitoneal repair performed using a subxiphoid top-down endoscopic totally preperitoneal approach (eTPA). Both procedures are relatively novel and represent alternatives to traditional intraperitoneal onlay mesh (IPOM) repair, whose use has declined due to concerns regarding mesh–viscera contact [6]. Available literature remains limited, consisting mainly of initial case series or indirect comparisons with IPOM [2–5]. In this context, the present study is among the first to provide a direct comparison between laparoscopic TAPP and ventral TEP, contributing evidence on their technical characteristics and short-term clinical outcomes.

In our analysis, operative time was longer in the TAPP group. This difference may be related to the need to create and close a peritoneal flap, a technically demanding step that can be particularly laborious when approached laterally from the left flank. Similar observations have been reported in early ventral TAPP series, where peritoneal flap management has been identified as one of the most challenging aspects of the procedure [7]. Conversely, mesh area was larger in the TEP group, likely reflecting the wider dissection enabled by pneumopreperitoneum, which allows broader mesh deployment in the preperitoneal plane. In TAPP, dissection is limited to the extent of the peritoneal flap, which may constrain mesh size, particularly on the contralateral side.

Another relevant technical difference concerns the management of associated rectus diastasis. In the TEP group, diastasis plication was performed when present, as the extraperitoneal midline approach from a subxiphoid access provides direct exposure and favourable ergonomics for midline suturing. However, diastasis closure was partial, since the subxiphoid access requires trocar placement at this level, which limits complete plication up to the xiphoid process. In contrast, in TAPP procedures, correction of rectus diastasis is technically more demanding and less ergonomic due to the lateral approach, which limits manoeuvrability and suturing along the linea alba. These findings suggest that ventral TEP may still offer technical and ergonomic advantages in patients with associated diastasis, despite the limitations inherent to the subxiphoid access, as also reported in recent series [2–5].

From a clinical standpoint, both groups demonstrated low complication rates without significant differences. Observed seromas and haematomas were self-limited and classified as minor (Clavien–Dindo I), in line with previously published experiences [3–5]. The absence of recurrence at 1 year is consistent with early reports showing low recurrence rates when the defect is closed and the mesh is adequately positioned in the preperitoneal space [3–5]. Hospital stay was short in both groups, with no statistically significant differences.

This study has several limitations. Its retrospective, single-centre design may introduce selection bias and limit generalisability. The small sample size reduces statistical power and precludes definitive conclusions. A minimum follow-up of 12 months may be insufficient to fully assess hernia recurrence, which can occur later; however, this reflects an early experience with a relatively novel extraperitoneal technique. In this context, outcomes may be influenced by the surgical team’s learning curve and by the evolution of surgical strategy over time, including a progressive transition between approaches while maintaining similar clinical indications. Accordingly, the results should be interpreted as preliminary and hypothesis-generating, rather than as evidence of superiority of one technique over the other.

## Conclusion

In this series, laparoscopic TAPP and ventral TEP approaches were safe and feasible for the repair of small and medium primary ventral hernias and W1 incisional hernias. The TAPP approach was associated with longer operative times, likely related to peritoneal flap creation and closure, whereas ventral TEP allowed wider preperitoneal dissection and the use of larger meshes. In addition, ventral TEP facilitated a more ergonomic midline reconstruction, enabling partial rectus diastasis plication in selected patients.

Given the retrospective design, limited sample size, and relatively short follow-up, these findings should be interpreted as preliminary and hypothesis-generating. Further prospective studies with larger cohorts and long-term follow-up are required to better define the role of each approach. Nonetheless, our results add to the growing body of evidence supporting extraperitoneal techniques as valid and increasingly relevant options in minimally invasive abdominal wall surgery [8, 9].

## Data Availability

The datasets presented in this study can be found in online repositories. The names of the repository/repositories and accession number(s) can be found below: https://doi.org/10.5281/zenodo.18521091.
